# Sleep disruption and its psychological treatment in young people at risk of psychosis: A peer methods qualitative evaluation

**DOI:** 10.1111/bjc.70002

**Published:** 2025-07-06

**Authors:** Felicity Waite, Steven Evans, Abreen Rebello, Tom Sharpe, Jummy Otaiku, Ellen Iredale, Thomas Kabir, Emma Černis, Daniel Freeman

**Affiliations:** ^1^ Department of Experimental Psychology University of Oxford Oxford UK; ^2^ Oxford Health NHS Foundation Trust Oxford UK; ^3^ NIHR Oxford Health Biomedical Research Centre Oxford UK; ^4^ McPin Foundation London UK

**Keywords:** at risk mental state, CBT, clinical high risk, cognitive therapy, insomnia, intervention, schizophrenia

## Abstract

**Objectives:**

A recent randomized controlled feasibility trial showed that sleep problems in young people at risk of psychosis can be successfully treated with psychological therapy and that this may bring additional benefits such as reducing depression, anxiety and paranoia. Here we report participants' qualitative experience of sleep problems and therapy.

**Design:**

A peer‐methods qualitative study employing reflexive thematic analysis.

**Methods:**

Semi‐structured interviews, co‐facilitated by peer researchers, were conducted with 16 young patients at risk of psychosis and having sleep problems who participated in the SleepWell Trial (ISRCTN85601537). Ten interviewees had received the 12‐week sleep therapy.

**Results:**

Four themes were generated: (1) the challenge to access mental health treatment (‘bouncing between services’), (2) sleep problems and mental health difficulties are intertwined (‘an obvious link’), (3) flexibility in therapy provision matters (‘tailored to me as a person’) and (4) improving sleep leads to wider benefits (‘fixing the sleep helped everything else’). Participants described a frustrating journey to access mental health treatment, marked by rejection and invalidation, which resulted in hopelessness and often resignation. The interaction between sleep disruption and other mental health difficulties was seen as obvious. Treatment for sleep problems was highly valued. The clear focus, therapeutic style and flexible delivery of the treatment was seen to create patient ownership, active engagement and hope. Participants described transformative changes: better sleep, fewer voices and fears and improved mood and confidence. Improving sleep made a difference to everyday life.

**Conclusions:**

Treating sleep problems in people at risk of psychosis is highly valued and often brings rapid and widespread improvements across a range of domains.

**Practitioner points**

- Young patients describe experiencing sleep disturbance as making other mental health problems worse and that this then exacerbates the sleep difficulties.
- Brief, targeted, psychological treatment for sleep problems is highly valued by young patients at high risk of psychosis.
- Improvements in sleep may bring benefits to mental health and other aspects of day‐to‐day life.

## INTRODUCTION

Sleep problems are a contributory causal factor to the occurrence of a range of mental health problems (Freeman et al., 2020), including psychotic experiences (Reeve et al., 2015; Waite et al., 2020). Successfully treating sleep problems brings wider benefits across mental health outcomes (Scott et al., 2021). In those at highest risk of a mental health condition, treating sleep problems may be a plausible preventative strategy.

Sleep problems are common both in patients with persistent psychosis (Freeman et al., 2019) and in young people at high risk for psychosis (Poe et al., 2017; Zanini et al., 2015), with estimates of prevalence in the at risk group of up to 75%. The range of sleep problems in patients with early psychosis is wide (Dondé et al., 2022). There are also high rates of comorbidity (Reeve et al., 2019). In qualitative accounts, patients describe disrupted sleep timing, anxious waking in the night due to nightmares or unusual experiences, and the negative impact on daytime functioning (Waite et al., 2018). Patients are eager for help (Waite et al., 2018). Yet few have received any treatment (Rehman et al., 2017; Stafford et al., 2024).

Sleep problems can be successfully treated with cognitive behavioural therapy (CBT) even in the context of other mental health conditions (Hertenstein et al., 2022). There are potential wider effects on a range of other mental health outcomes (Freeman et al., 2017; Scott et al., 2021). CBT for sleep problems has been successfully adapted for people with diagnosed psychotic conditions such as schizophrenia (Freeman et al., 2015; Sheaves, Freeman, et al., 2018; Sheaves, Isham, et al., 2018). Given the wider benefits of treating sleep problems, it is also plausible that intervening on sleep before the onset of psychosis might be preventative (Bradley et al., 2018; Freeman et al., 2017, 2020). This is a stance that is consistent both with a network perspective (Borsboom & Cramer, 2013) and a clinical staging model (McGorry et al., 2014) in which common non‐specific factors, which are causally connected within dynamic networks of interacting symptoms, may act as early treatment targets to reduce the likelihood of severe mental health problems. This is what we set out to begin to test in the SleepWell trial (Waite et al., 2023).

In our feasibility trial, 40 young patients (aged 14–25 years old) at ultra‐high risk of psychosis were randomized to receive our eight‐session targeted psychological treatment (SleepWell) in addition to usual care or to continue their usual care (the control condition). Compared to usual care, provision of the sleep therapy led to substantial reductions in sleep problems and wider benefits across mental health outcomes (Waite et al., 2023). The treatment was popular: 95% of patients took up the treatment, and there were high acceptability scores. There were very large effect size improvements in sleep post‐treatment (Cohen's *d* = 2.7) which were maintained at the 9‐month follow‐up (*d* = 1.9). Provision of the sleep therapy was also associated with medium effect size improvements in anxiety, depression and paranoia, with larger effects observed at the later follow‐up.

In this study, we used a peer research approach to try to gain an in‐depth understanding of the experience of sleep problems and their treatment with the SleepWell therapy. Peer research is designed and conducted by people with relevant lived experiences. Peer methods can be seen as an important shift towards partnership in the research process (Prebeg et al., 2023). Employing peer methods has the potential to facilitate more nuanced and rich data collection and analysis by enhancing rapport (Terry & Cardwell, 2016) and levelling power (Harding et al., 2010). This collaborative approach, including researchers with different perspectives and expertise, including lived experiences, can strengthen the validity and trustworthiness of qualitative methods (Gillard et al., 2010; Sweeney et al., 2013). Reflexive thematic analysis (RTA; Braun & Clarke, 2021) is an interpretative approach to qualitative data analysis that facilitates the identification of themes (recurring concepts or ideas) in the data and explicitly acknowledges and emphasizes the active role of the researcher in the interpretation. This approach complements peer methods and enables a combination of peer and non‐peer perspectives. We conducted a peer‐methods qualitative study, employing RTA, to explore SleepWell trial participants' experiences of sleep problems and their treatment.

## METHODS

### Design

The study received ethical approval from the Health Research Authority (HRA/HRCW: IRAS 281235, The SleepWell trial) and NHS South Central—Oxford A Research Ethics Committee (20/SC/0281) as part of the SleepWell trial.

We conducted a peer‐methods qualitative study. The study was designed, conducted and analysed in collaboration with people with relevant lived experience. This included three peer researchers from the SleepWell Lived Experience Advisory Panel (LEAP). The SleepWell LEAP is a group of four people with lived experience of sleep problems and psychotic‐like experiences. The LEAP was facilitated by the McPin Foundation (www.mcpin.org), a charity that exists to put the lived experience of people with mental health problems at the heart of research. In line with guidance set out by Prebeg et al. (2023), there was a degree of flexibility (i.e. there were different types of involvement opportunities and roles: advisory panel, data collection, data analysis, authorship) and fluidity (i.e. the opportunity and support to facilitate movement between roles: training on data collection and analysis) in the peer methods. There was also an active effort and attention given to the degree to which power was shared within the research team and process (relational empowerment).

Reflexive Thematic Analysis (Braun & Clarke, 2021) was used to analyse the data. This analytic method is used to identify and infer patterns of meaning across a data set. A critical realist approach was taken based on the assumption that any objective reality is observed through the unique lens of the researcher. Therefore, this method can accommodate combining peer and non‐peer researcher perspectives. Criteria to promote credibility in qualitative research were used to inform the design and conduct of the study (Braun & Clarke, 2021; Tong et al., 2007)—for example Table S3.

### Research team—Positionality statement

People with lived experience of sleep problems and psychotic‐like experiences were involved in all stages of the study. The research team included three peer researchers who have had similar experiences to those being explored and clinical psychologists who developed the sleep therapy. Working with the LEAP, we aimed to use peer knowledge in the design, procedure and presentation of findings.

### Participants

Participants had taken part in the SleepWell trial (ISRCTN85601537; Waite et al., 2023). The participants were young people (aged 14–25 years) attending NHS mental health services with sleep problems who are at ultra‐high‐risk of psychosis (assessed on the Comprehensive Assessment of At Risk Mental States, CAARMS) (Yung et al., 2005). The inclusion and exclusion criteria for the trial are listed in the trial protocol (Waite et al., 2020). The additional criteria for this qualitative study were: willing and able to give informed consent and willing to have the interview audio recorded and for the use of pseudonymized verbatim quotes. Criterion‐based purposive sampling was used to maximize variation in participant characteristics including uptake of therapy, age, gender and ethnicity. Participants were invited to take part in the qualitative study after their final follow‐up assessment in the trial (9 months after the baseline assessment). We aimed to recruit 16 people who had taken part in the trial: 10 who had been offered the therapy and six who had not (i.e. were in the treatment as usual only control group). Seventeen people were invited to participate, one declined, 16 consented. Written informed consent, including consent to the use of pseudonymized quotes, was obtained from all participants. Participants were reimbursed for their time.

### The intervention

The SleepWell therapy is a brief psychological intervention designed for young people (14–25 years) and targets three mechanisms that help to regulate sleep: circadian rhythm, sleep pressure and hyperarousal. To address circadian alignment (the timing of sleep) we re‐align sleep–wake cycles with the natural night‐day cycle, using light as the key zeitgeber (time cue). We also use morning routines, daily activity timepoints (i.e., a specific activity at a set time), social connection and mealtimes to support circadian entrainment. Given the common shift in adolescence to a delayed‐phase circadian pattern, additional emphasis was put on establishing a consistent sleep window (the ideal time period the person would like to be asleep) and then adjusting the timing of this window to align with the young person's ideal sleep time. To address sleep pressure (the propensity or need for sleep) we promote daytime activity, including the use of fitness trackers. To address hyperarousal, the key strategy is stimulus control, in which patients learn to re‐associate bed with sleep. We also use strategies to reduce worry and promote night‐time relaxation.

The SleepWell intervention is manualized in a modular format: modules can be selected by patients on the basis of individual need, enabling the intervention to be personalized. The format and manuals were developed in collaboration with the LEAP. Measurement of the sleep problem is taken in every session in order to track progress and retain focus. The treatment was informed by standard CBT sleep protocols (Espie, 2006; Harvey, 2002) and protocols for treating sleep disruption in patients with psychosis (Sheaves, Isham, et al., 2018; Waite, Myers, et al., 2016) with specific adaptations for young patients at high risk of psychosis, such as addressing psychotic experiences or external stressors (e.g., living independently for the first time or taking exams) that affect sleep and navigating environmental constraints (e.g. shared accommodation with siblings or at university).

The intervention was delivered on an individual basis, in approximately eight 1‐h sessions over a 12‐week period by a clinical psychologist. Contact between sessions, such as brief telephone calls, text messages or email, was provided to promote the implementation of treatment techniques. Treatment sessions were conducted at the patient's home, at an NHS clinic, or via videocall.

### Interview guide

The semi‐structured interview guide (see Data S1) was developed with reference to existing literature, guidelines for good practice and in collaboration with the LEAP. It was further refined following pilot interviews with the LEAP. There were three main topics: the participants' experiences of sleep problems; any changes in sleep over the period of trial participation; the experience of taking part in the study and, if applicable, the experience of the sleep therapy. Following guidance from the LEAP, the interview guide was provided to participants before the interview. During the interview, it was used flexibly to focus on the participants' narratives and allow for the exploration of new ideas. Tailored prompts were used to elicit further detail, clarify points, and refocus if needed. The participants' own language was used whenever possible. All four interviewers had received training in interview methods, and each practised the interview in a series of pilots.

### Interview process

Interviews were conducted remotely (in line with COVID‐19 restrictions) between February 2022 and January 2023. Each interview was facilitated by one peer researcher (AR = 9, SE = 7) and one clinical psychologist researcher (EČ = 4, FW = 12). At the start of the interview, the researcher and peer researcher introduced themselves, their expertise and relevant experience. After this, the peer researcher chose when, how and what to share with the participant about their potentially related experiences. This differed between interviews. For six participants, the clinical researcher facilitating the interview delivered the sleep therapy. The peer researchers were not known to any participant. Interviews were conducted in a single session; the duration was between 17 and 68 min (mean = 42 min, SD = 17 min). Brief field notes were taken. Interviews were audio‐recorded, transcribed verbatim and deidentified.

### Analysis

The six‐phase process outlined by Braun and Clarke (2006, 2021) was followed. This included (1) familiarization with the data; (2) generating initial codes—both semantic and latent; (3) generating themes; (4) reviewing potential themes for coherence, meaning and robustness; (5) defining and naming themes and (6) producing the report. Throughout the analysis, specific consideration was given and efforts made to incorporate the range of ways people communicate and articulate themselves during qualitative research (Gupta et al., 2024).

The analysis was completed by multiple coders, all trained in data analysis, including two peer researchers (SE and TS). This was undertaken in a collaborative, reflexive approach with the aim of achieving richer interpretations of meaning, rather than seeking consensus. The analysis was an iterative process with themes discussed with the LEAP and co‐authors to consider the most appropriate way to convey the participants' experiences. For example, setting the context of the sleep therapy within the challenging journey to access support was important to the LEAP and is therefore presented as the first theme to provide the reader with this perspective.

398 references were coded. These initial codes were organized into three themes in which common ideas were clustered. The themes were reviewed, reconceptualized and re‐labelled to produce the final four themes and three subthemes reported.

### Reflexivity and credibility

Prior to data collection, bracketing interviews were conducted to help understand the positionality of the team. The interviewers each maintained a reflexive log throughout the data collection process and had a brief period of joint reflection after each interview to note key learning, draw on the two perspectives and identify any oversights. The construction and interpretation of the coding and themes were discussed and refined with the LEAP and co‐authors to invite different perspectives.

## RESULTS

### Contextualizing the findings

Sixteen people took part. The average age was 18 years old (SD = 2, range 15–22). Eight participants identified as male, seven as femaleand one as other. Most participants were White (*n* = 13), two participants were Asian and one was mixed heritage. Ten participants had received the SleepWell intervention (expected to be delivered in around 8 sessions, with a treatment dose defined as at least four sessions). The mean number of sessions attended was 8.7 (SD = 1.9, range 6–12). All participants had experienced sleep problems. Assessed using the Insomnia Severity Index (ISI) the mean score for all 16 participants at the start of their participation in the trial was in the moderate insomnia range (mean = 17.8, SD = 1.9, range 11–23). For the 10 participants who received the intervention, the mean baseline score was 18.8 (SD = 3.16, range 12–23). At the 9‐month follow‐up assessment (conducted approximately 6 weeks before the interview), the mean score for the 10 participants who had received therapy was below the clinical cutoff for insomnia on the ISI (mean = 6.5, SD = 5.4, 0–17). For the six participants who did not receive the intervention, the mean score on the ISI was 16.1 (SD = 3.43, range 11–21) at baseline and 11.8 (SD = 6.6, range 6–20) at the 9‐month follow‐up assessment. The participant characteristics in this qualitative study, including changes to sleep problems post‐treatment, are consistent with those seen in the main trial across the whole patient group (see the trial outcome report; Waite et al., 2023).

### Overview

Four themes were generated: (1) The challenge to access mental health treatment (‘bouncing between services’), (2) sleep problems and mental health difficulties are intertwined (‘an obvious link’), (3) flexibility in therapy provision matters (‘tailored to me as a person’) and (4) improving sleep leads to wider benefits (‘fixing the sleep helped everything else’). Participants described being overlooked and finding it hard to obtain help—for sleep problems as well as other mental health difficulties. This left patients with a sense of isolation, frustration and hopelessness. Participants described being engulfed in a loop of co‐occurring sleep disruption and mental health struggles. For those who received the SleepWell intervention, flexibility in the delivery of the treatment enabled participants to actively and effortfully, make progress and take ownership of the changes and the process. There were many benefits beyond better sleep: fewer voices and fears, improved confidence, reduced suicidal thoughts, greater connection with others, attendance at work and college and engagement with everyday life. Additional illustrative quotes are provided in Table S1. The pattern of participant contribution to the themes is shown in Table S2.

### Theme 1: The challenge to access mental health treatment: ‘Bouncing between services’

Participants described the repeated challenges of accessing help—for sleep problems as well as other mental health difficulties: ‘Obviously, people like me, obviously have gone through the cracks, a lot’ (P3). They described being overlooked and finding it hard to obtain help. The language participants used was striking: ‘laid off’ (P6), ‘abandoned’ (P14), ‘ignored’ (P11) and ‘didn't want me’ (P3). There was a clear sense of rejection and frustration:

Yeah, so uh, when I went into services, they kind of just, swiped me under the rug and said ‘You know look, we deal with people who are upset or anxious or, kind of this’ and they literally said to me ‘You're too much of a problem for us with all of the stuff that you're doing to yourself and all the stuff that you're going through so what we're going to do is, we are going to treat it nowhere near as seriously as it should've been' uhm, and you know for me back then, it kind of hit a bit.’ (P6)

Here P6 describes their experience of being ignored, invalidated and uncontained as they sought help from mental health services. There is repetition throughout the interview of key phrases, for example, ‘I'm gonna be honest, they weren't very good with me. They kind of, they kind of laid me off and said I was too much of a problem for them to deal with’. As with many participants, this knocked the participants' confidence in services and in themselves—making it harder to continue the journey to accessing help.

These challenges left patients with a sense of isolation and resignation at the start of therapy, rather than hope or expectation. With participants using phrases such as: ‘nothing to lose’ (P9), ‘worth a shot’ (P11), ‘may as well’ (P13), ‘this could be a big waste of time’ (P3) and ‘try anything’ (P16).

The LEAP flagged the potential longer‐term consequences of not intervening at this key time of mental health deterioration which often occurs during an important developmental life stage. They highlighted the potential benefit of a brief sleep intervention: ‘it could make all the difference for those on the edge’ and identified opportunities to embed this approach within the treatment pathway between primary and secondary mental health services. This suggestion was consistent with participant accounts in which the sleep treatment was considered a first step, a route to build trust and confidence in services.

### Theme 2: Sleep problems and mental health difficulties are intertwined: ‘An obvious link’

Participants described the interaction between sleep disruption and mental health difficulties as ‘obvious’ (P11). Noting with confidence, for example, ‘100% definitely, definitely, definitely’ (P6), that sleep problems, anxiety, depression and psychotic‐like experiences were ‘intertwined’ (P10). The consequences of the spiralling sleep problems and other mental health difficulties were significant: knocking participants' confidence and energy to engage in everyday life, with the LEAP members noting how this often led to isolation, which bought further negative impacts on mental health.

Further to this, participants spoke of the complexity of the sleep disruption itself. Sleep was unpredictable. Sometimes there was too much sleep, sometimes too little and at other times no sleep at all:

So, in the day, I was just so overwhelmed and anxious by everything, that all I wanted to do was just sleep. But then, sometimes, it would be the opposite. I would be so anxious and worked up that I just can't sleep at all, or I will go to sleep and wake up anxious. Go back to sleep, wake up anxious and in a cycle. So, it's not always that I can't sleep but sometimes I think I just want to sleep all the time if I'm particularly anxious, which then, I guess, if I'm sleeping in the day because I'm anxious, then at night you can't sleep because I've slept all day. (P13)

#### Sub‐theme: Bidirectional interaction between sleep problems and psychotic experiences

A bidirectional relationship was described between psychotic experiences and sleep problems: sleep disruption could both cause voices and fears and result from those psychotic experiences:

So, if I hadn't slept well, I would end up hearing things a bit more, end up seeing things a bit more and it ended up just really, being worse off without sleep. I think it's something to do with the fact … like I said before, your brain gets overworked, and it starts to like … in terms of computer terms, it starts to malfunction, and it would start to, like, deteriorate a little bit. Then when you sleep, it starts to repair itself. It's like fixing a computer with viruses in it. When it shuts down and it sleeps, it works … it starts to work very well the next day. And I think of the brain like that, as in it's like a computer. As in, it has to shut down, reboot and then it can start doing its daily process. (P10)

It was definitely the shadowy figures and those voices that got in the way of sleep, because it just made me anxious. So, I stayed awake for a long time, just so I could calm down. I used to go on games and distract myself instead of having to look at myself. It was tiring. I had like low confidence, low mood and low self‐esteem, and during the day I was tired, and it meant I couldn't function properly. (P9)

For those participants who described being wary and suspicious of others, disrupted sleep made it even harder to trust: ‘I'm exhausted maybe I don't want to see any good in someone, so that will make me less likely to trust them’. (P16) and these concerns often played on their mind at night, making it harder to sleep.

For many participants, the interplay of sleep disruption and mental health difficulties was experienced as overwhelming and inescapable. With the interaction described as ‘leading me down a hole’ (P6), a ‘downward spiral’ (P16), a ‘negative loop’ (P6, P9) or ‘cycle’ (P13) that ‘engulfed’ (P8) and ‘trapped’ (P14) participants. The LEAP members reflected how this was consistent with their experiences, as one member put it: ‘both the spiral down and recovery can be defined by how you are sleeping’.

### Theme 3: Flexibility in therapy delivery and provision matters: ‘Tailored to me as a person’

Throughout the participant accounts, the importance of personalization was clear. The specific style of the therapy—characterized by a collaborative, focused, tailored approach—allowed patients to feel heard, validated and empowered to take action and then create a sense of self‐ownership of the progress made. Two subthemes are reported that highlight the dynamic interaction between (a) the therapy style, content and deliverer and (b) the active, effortful engagement by the patient.

#### Sub‐theme: Flexibility and rhetoric matter

As reported in Theme 2, participants reported many interacting difficulties that felt overwhelming and unstoppable. In contrast, the therapy had a clear target: improving sleep. This provided focus amidst complexity. The clear aims of the therapy were reflected in the name of the approach, ‘SleepWell’. The ‘straightforward and transparent’ (P8) language helped foster trust. The rhetoric, as termed by the LEAP, of the approach was important for promoting hope, and an understanding of what was expected in this active approach: ‘Not talking about stuff, actually doing stuff’. (P1).

Choice, tailoring and personalization were seen as embedded in the approach, and were highly valued. The selection and application of techniques was experienced as individualized: ‘it was very tailored to me as a person’ (P6). With further recognition that it was personalized for all: ‘I genuinely felt like every single person that was a part of this was cared for and thought of and every single case had been adapted to fit that person’. (P14). Yet at times personalization required contrasting styles of treatment delivery. For example, one participant noted that for neurodivergent participants, flexibility meant consistency of sessions and reducing the social demands of the sessions: ‘Just about flexibility around a person's different issues, like disabilities or mental illness and stuff. Like, if they have Autism, maybe slow down and let the person think more, rather than being quick … it was difficult for people like me, how to react and how to plan in advance for sessions. … but I think it's good because being active in therapy [it] helps you talk better and let things out’ (P9). Therefore, there was choice in the practicalities of sessions, for example, the location, modality (online or in person, remaining within the therapy room or taking a walk during the session), timing and use of materials.

Accessible explanations of the techniques and tailored rationales for their use boosted uptake, for example: ‘I found it quite easy to follow. They kind of adapted to my situation, they were very good at that. So, most of the tasks was easy to complete’. (P16). There was a spirit of curiosity and experimentation with the therapy techniques—working collaboratively to discover how a strategy might work best for the individual. Prompts and communication between sessions supported people to try out the techniques: ‘they were good with communication, with like emails and text messages to help remind me, so they definitely helped with the reminders of things’ (P16). Regular measurement of key outcomes—shared using summary graphs in sessions—provided a ‘massive eye opener’ (P14). Individuals often described the powerful gains made from specific techniques and shared their understanding of how they might work, for example the wind‐down routine was described as: ‘having that time to cool down gave my brain a bit of quietness to fully chill out’. (P8). The focus, language and tailored techniques were provided in the context of a therapeutic stance in which participants described feeling heard, understood, remembered and therefore cared for

Remembering about the people you're talking to definitely made me feel more comfortable because I felt listened to and acknowledged. And for me, the ability to see progress was great. Not just in myself but see it on paper; in graphs and reports that had been built up over the many weeks. To see that result was very, very positive for me to physically look in front of me and go, “cool, here is the actual black and white of how I felt and then this is now”. That was really positive. And having the ability to work on it yourself and fill out things, your own personal experience. And then work on that in sessions was great. (P8)

This extract reflects the specific features of the style, content and delivery of the therapy which were important to participants. These features helped the person to make successful progress and learn skills that could be applied over the longer term. For some, the end of therapy was experienced as abrupt, it ‘suddenly stopped’ (P9), for others, it ‘gradually fizzled out’ (P1). Some participants described a sense of loss following a meaningful therapeutic connection and long fought for support. Sometimes it was hard to sustain change after the regular sessions finished: ‘so I started to slip up’ (P9). Others noted a mix—both satisfaction and nerves at the end of therapy—reflecting the important dynamic of collaboration in therapy and independently driving and owning progress: ‘so I think ending it was … it was nice to know that there had been an end point that had reached, and a result was there, but still a little bit anxious to be on my own with it and not have that person to, like, talk to and discuss about the issues, I suppose.’ (P8).

#### Sub‐theme: Active engagement and ownership: Making the most of therapy

Participants described their active effortful approach to the therapy with a sense of ownership: ‘I did the hard work’ (P3), ‘I put the effort in’ (P6). Participants were eager to prepare, learn and make the most of the sessions. The therapy materials facilitated a process of discovery—gaining new knowledge and insights. There was an impressive level of detail in the description of specific techniques. One participant described using their new expertise to support their friends to also achieve better sleep. The refreshing, practical, interesting therapy was hard work, yet enjoyable. In the extract below the participant describes how they became increasingly self‐reliant as they now had the necessary skills and knowledge to improve their sleep and wider day‐to‐day life:

I don't know how to explain it … it was nice to be able to rely on myself to do the routines and everything else. So yeah, it was just very nice to be able to rely on myself instead of everybody else to tell me how to do it. It was relieving that I now had the solution to my problem. (P11)

Despite the challenging journey to access help and the sense of resignation described in Theme 1, participants became actively committed and engaged in the therapy, finding a ‘willingness and wantingness to do it’. (P16) as they became self‐reliant and able to successfully apply their learning over the longer term, as it became ‘just quite normal now’ (P7).

The flexibility, rhetoric and individualization of the therapeutic approach was highly valued by all participants in the study. There were also clear descriptions from interviewees who did not receive the therapy of similar helpful features of how the trial was conducted, such as the choice and options that were actively provided throughout the consent, randomization and assessment procedures.

### Theme 4: Improving sleep leads to wider benefits; ‘Fixing the sleep helped everything else’

The final theme reflects the meaningful changes participants described as a result of the intervention and the resulting improvement in sleep. This included both the scale of the change, often described as life‐changing, and the breadth of the ‘knock on benefits’ (P6, P8), all of which were said to be noticeable to others.

One of the things that was rough before was sleep and I think that's sort of had a knock‐on effect with sort of everything else to do with my mental health. But the sleep, since being in the trial, has improved drastically and it's sort of, again, I've said it had a knock‐on, in a positive way to sort of stabilise everything. (P8)

Participants used similar evocative phrases when describing the scale of the changes, such as ‘transformative’ (P3, P14), ‘massive difference’ (P16) and ‘it changed my life’ (P2, P10 and P14). Participants particularly noted their surprise at the amount of change achieved in such a short time frame, and when taking a specific focus on one of the many challenges, they were facing: sleep disruption. The sense of transformation was often linked to the wide range of benefits that participants had noted:

it's just been a snowball effect of taking more things in and being able to step back a bit more and that's what the therapy has really done for me. It's really allowed me to look at things a lot more, the way I dress, the way I hold myself, the way I act around certain people, the way I interact with people I don't know. People with authority, people above me at work, people‐‐ everybody, everybody and everything I now can sort of see‐‐ I can take a second and look and think before acting. I now feel like everything I do with purpose. And I have the therapy to thank for that, that is sort of unlocked that ability for me. And I really do think it is just patience. That's what it's unlocked for me. (P14)

In this extract from P14, wider changes beyond improving sleep were described as a ‘snowball effect’—reflecting the sense of momentum and ever‐growing impact. For this participant, despite the specific focus on improving sleep, the therapy helped change their perception of themselves and their abilities, giving confidence in their capacity to handle situations. This potential was within the participant but hard for them to access, and the brief therapy helped them to ‘unlock’ patience, perspective and purpose.

The most commonly described benefits were better sleep, fewer voices and fears and re‐engagement with everyday life, for example: ‘seeing things and hearing things has pretty much disappeared. I'm gonna be honest. … Sleep and stress could always cause this stuff but it just started filtering away and of course the less it happens the less you realise that it happens, um, and its been so long since I've had any like noticeable episodes I guess um, that is just, its just kind of gone’ (P6); ‘I think sleeping has improved my mood, I wake up happier and I think that sleeping better and being able to sleep faster in the, at night has lowered, you know, my voices at night’ (P15). Sleep disruption was ‘fixed’ (P15), and the benefits were tangible. Participants spoke of how anxiety and low mood improved, as well as a new‐found level of confidence which allowed them to do things that previously felt out of reach, for example: ‘Yeah, it helped me boost my confidence so I could do more things like go to the shops, go to other places which I wouldn't normally go. Because normally I wouldn't sit on a bus or sit on a train on my own because I'd feel scared and I wouldn't do those things but over time I managed to do both things’. (P9). Improvements in energy levels and greater connection with others were often linked to improvements in daytime activity, as people began to re‐engage in work, college and the activities that matter to them. There were other wider improvements such as being able to think clearly, less irritability, fewer suicidal thoughts, fewer appearance‐related concerns and better physical health. For the wider benefits participants described potential mechanistic routes to explain the pattern of change: noting that improved energy allowed re‐engagement in activity, waking refreshed protected against low mood, thinking more clearly allowed more problem solving, and better sleep not only reduced stress in itself but helped give flexibility, patience and confidence to cope in stressful moments. For some participants, there were fewer changes, for others, the changes were hard to sustain. Overall participants described greater ease, energy and engagement in life.

## DISCUSSION

In this peer methods qualitative study, we set out to understand the experience of sleep disruption and its treatment in young people at risk of psychosis. Participants were eager for help to improve sleep and mental health, despite a challenging journey to access support. Participants spoke of an obvious cycle of sleep problems and other mental health difficulties, including psychotic experiences, exacerbating each other. Treatment for sleep problems was highly valued. Consistent with the outcomes of the trial, the participants described rapid and wide‐ranging benefits that persisted over time. These results support the assertion that treating sleep problems can bring quick mental health benefits. Whether intervening on sleep can have more preventative mental health benefits for young people at risk of psychosis will be looked at within the new large multi‐centre Sleeping Better trial (ISRCTN71800376).

There was further consistency with the quantitative findings (Waite et al., 2023): the treatment was popular, there were large changes in sleep, and participants described improvements in anxiety, depression and psychotic experiences. The transformative benefits described by several of the participants in our study are consistent with the outcomes of a series of trials across the spectrum of severity of psychosis, all of which demonstrate large effect size improvements in sleep, very high uptake of therapy, and indications of the potential wider mental health benefits (Freeman et al., 2015, 2017; Sheaves, Freeman, et al., 2018).

The findings in this study are also consistent with other patient accounts of sleep disruption in the context of psychotic experiences, including: the complexity of sleep problems and range of presentations (Waite et al., 2018), the wide impact of sleep problems (Chiu et al., 2016; Faulkner & Bee, 2017), and the value and benefits of treatments for sleep disturbance (Waite et al., 2018; Waite, Evans, et al., 2016). Both the sense of hopelessness at the outset of treatment and the eagerness for help is common in accounts from patients with persistent psychotic experiences (Chiu et al., 2016; Faulkner & Bee, 2017; Waite, Evans, et al., 2016) and those at risk of psychosis (Waite et al., 2018), including an openness to the use of technology in treatment (Taylor et al., 2022), such as wearable devices (Griffiths et al., 2021). To our knowledge, this is the first peer methods study to explore first person accounts of sleep disruption and its treatment in young patients at risk of psychosis. The peer researchers gave a particular opportunity to discuss experiences of therapy, as well as difficulties with sleep and other mental health problems, and bought this specific lens to the analysis. These findings provide an insight into the dynamic process of therapy in which the specific style, content and alliance enabled participants to take both an active role and degree of ownership of the resulting improvements. The importance of personalization and tailoring was clear—with further work to adapt for neurodivergent participants required. Personalization may be particularly important in future implementation, especially given the complexity of the presentation of sleep problems in this group (Dondé et al., 2022; Freeman & Waite, 2025; Harvey, 2022; Reeve et al., 2019). There are promising initiatives to embed sleep interventions into youth mental health services (Rollinson et al., 2024), and potential opportunities within the NHS community transformation initiative. Given the challenges and evocative language described in the first theme, as well as the potential window of opportunity for prevention, it is imperative that any intervention in this high‐risk group is accessible.

There are a number of limitations. Firstly, the research was conducted in a single centre in the United Kingdom and the participants will not be representative of all young patients attempting to access services. Notably, there were no specific clinical services for young people at risk of psychosis at the recruiting sites; therefore, usual care was limited. There was limited ethnic and gender diversity in the participants interviewed. Differential access to mental health services as well as treatment outcomes for psychosis are reported in young patients from minoritized backgrounds (Chui et al., 2021; Coelho et al., 2022; Morgan et al., 2017), especially Black African and Black Caribbean patients. Therefore, understanding the potential barriers faced by underrepresented groups will be important in both future evaluations of the therapy and implementation. The sampling method was intended to maximize variation, including with regard to therapy uptake; however, we only interviewed people who had completed therapy, which is largely a reflection on the uptake of the therapy in the trial (only one person did not receive a treatment dose). There may be specific challenges for those participants who choose not to complete therapy, which will be more likely to be seen in larger trials. Similarly, there were extremely few people in the trial who did not improve their sleep from the intervention, and their perspective will likely be different and was not captured in the current report. Therefore, one notable omission from the data set is detailed insight into the potential challenges and drawbacks of therapy. This is potentially due to sampling bias—as outlined above. It may also be due to difficulties in data collection. Peer methods were used to try to balance power and invite rich accounts. However, a clinical psychologist researcher was also present. For a subgroup of participants (*n* = 6), this was the therapist they had worked with in the trial intervention. In recognition of this limitation in the data, a LEAP meeting was held specifically to consider the potential downsides and drawbacks of therapy. The LEAP highlighted the investment of time and resources and the potential emotional demands of a psychological intervention. Future work is needed to investigate potential adverse effects and demands of psychological interventions in young people at risk of psychosis. Working within a peer methods framework, we deliberately sought out different perspectives, experiences and expertise throughout the data collection and analysis. However, we recognize the persistence of an ableist bias within our methods, common in qualitative research with patients with psychosis (Gupta et al., 2024), reflected in the use of articulate illustrative quotes within the report. In line with recent recommendations (Gupta et al., 2024), we want to find routes to improve inclusivity with regard to neurodiversity and the range of ways people articulate and communicate. It would also be valuable to seek further feedback from the peer‐researchers regarding their experience of actively engaging as partners in the research process. In other peer research with young people at ultra‐high risk of psychosis, the role has been highly valued (Trimmel et al., 2024). Understanding the peer experience in terms of flexibility, fluidity and relational empowerment (Prebeg et al., 2023) could inform future peer‐methods research. We also note that the therapy was conducted during the COVID‐19 pandemic. The context of restrictions and disruption, within services and day‐to‐day life, may have affected the experience of sleep disruption, mental health difficulties and the therapy.

Young people at risk of psychosis face multiple mental health problems. Given sleep disruption is a contributory causal factor in the occurrence of mental health difficulties, treating sleep problems may be a valuable starting point for intervention—it provides focus amidst complexity, is quickly tractable and may bring wider benefits to both the short and longer‐term trajectory. The next step is a definitive trial, with embedded mediation and moderation tests, to determine the wider effects. The Sleeping Better trial (ISRCTN71800376) is now underway.

## AUTHOR CONTRIBUTIONS

FW was the chief investigator, conceived of the study, had full access to all the data and wrote the first draft of the manuscript. FW and DF led the trial design and treatment design. TK led lived experience involvement. The SleepWell Lived Experience Advisory Group (SE, AR, TS and JO) provided lived experience expertise. EI recruited participants. SE, AR, EČ and FW conducted the interviews. SE, TS and FW led the analysis. All authors contributed to critical review and editing of the manuscript.

## FUNDING INFORMATION

The trial was funded by the NHS Research for Patient Benefit (RfPB) programme (Grant Reference Number: NIHR200481). It was also supported by the NIHR Oxford Health Biomedical Research Centre (BRC‐1215‐2000/NIHR203316). FW is supported by a Wellcome Trust Clinical Doctoral Fellowship (award number 102176/B/13/Z). DF is a NIHR Senior Investigator. The views expressed are those of the authors and not necessarily those of the NIHR or the Department of Health and Social Care.

## CONFLICT OF INTEREST STATEMENT

All authors declare no competing interests.

## Supporting information


**Table S1.** Additional illustrative quotes.

**Table S2.** Summary of participant contributions to each themes.

**Table S3.** 15‐point checklist for good practice in Reflexive Thematic Analysis (Braun & Clarke, 2021).

**Data S1.** Supporting Information.

## Data Availability

The data that support the findings of this study are available on request from the corresponding author. The data are not publicly available due to privacy or ethical restrictions.
