# Supplementary material for: Sleep disruption and its psychological treatment in young people at risk of psychosis: A peer methods qualitative evaluation
Source: Br J Clin Psychol. 2025 Jul 6;65(3):746–61. doi: 10.1111/bjc.70002 (PMC13456408; doi:10.1111/bjc.70002)
Supplement: Supplementary file 1 [file BJC-65-746-s002.pdf]

Experiences of taking part in the SleepWell study

Participant copy of questions

The interviewer(s) will introduce themselves and remind you about the SleepWell trial, tell you how long the interview will be (and let you know about breaks etc.), and will check again that we have your permission to record.

The interviewer will give you a short update on how the study is going at the moment before we begin the interview.

We will then discuss these main topics:

1) **How are things for you at the moment, and your sleep in particular?**

2) **Have there been any changes?**
In your sleep? In other areas? Has anyone else (friends or family, or other significant or important people important in your life) noticed anything has changed?

3) **What was the study like? (How did you find it taking part?)**
e.g. what were the assessments like? What was it like to have 50:50 chance of getting the full therapy? Etc.

3a) What was the impact of the COVID-19 pandemic on your experience of taking part in SleepWell?
e.g. what was it like having remote sessions, or researchers wearing PPE? Etc.

4) ***If you had it:* How did you find the therapy?**
e.g. what was most / least helpful? What was it like working with your therapist?

5) **Is there anything that we haven't asked that you feel may have been important to your experience?**
Any suggestions for a future version of the study? Anything else you would like to add?

If there are any comments or opinions you would like to feedback but forget to mention in the interview, or do not feel able to say during the appointment, you can send these to the SleepWell team anonymously using this online comment box:
