# Supplementary material for: Sleep disruption and its psychological treatment in young people at risk of psychosis: A peer methods qualitative evaluation
Source: Br J Clin Psychol. 2025 Jul 6;65(3):746–61. doi: 10.1111/bjc.70002 (PMC13456408; doi:10.1111/bjc.70002)
Supplement: Supplementary file 2 [file BJC-65-746-s001.docx]

Supplementary table 3: 15-point checklist for good practice in Reflexive Thematic Analysis (Braun and Clark, 2021).

| **Process** | **No.** | **Strategies undertaken** |
| --- | --- | --- |
| **Transcription** | 1 | The data were transcribed verbatim, checked for accuracy against the audio recordings by the transcriber, and two researchers. |
| **Coding and theme development** | 2 | Each transcript, code, and proposed theme was reviewed repeatedly in an iterative process. Reflections regarding the reactions, emotional responses, and meaning of each section of data were noted in the reflexive log. |
|  | 3 | Thorough, inclusive, and comprehensive consideration was given to each transcript, with awareness of the range of ways people communicate (i.e. caution regarding excessive reliance or influence of articulate and vivid examples). A curious stance was adopted involving an assumption that the researcher required an active reflection to seek to understand the participants’ experiences. |
|  | 4 | All relevant extracts for each theme were collated and reviewed during a LEAP meeting. |
|  | 5 | Candidate themes were checked against coded data, the original dataset, and for coherence with peer researchers and other members of the research team. Themes were revised at each stage. |
|  | 6 | Each theme was reviewed by the LEAP, supervisor, and wider research team to ensure internal coherence, consistency, and distinction. Each theme is organised around a central concept with sub themes used judiciously to provide nuance that was particularly meaningful to participants (Theme 2), and to reflect the dynamic interaction of two positions within a central concept (Theme 3). |
| **Analysis and interpretation** | 7 | Data have been analysed and interpreted using peer methods. This includes data collection and direct coding of transcripts, as well as discussions in response to presentations of potential themes and example data extracts. The theme titles reflect the analytic process: they provide the active interpretation of the data by the research team as to what is meaningful to participants. |
|  | 8 | There is consistency between the analysis and data, with extracts used to evidence the analytic claims, with further emphasis noted when the analysis resonated with particular components of the peer researchers’ lived experiences. All participants provide extracts in the written report and their contribution across themes is summarised in Table 3.2. |
|  | 9 | The analysis addresses the aims of the research and provides a narrative of participants’ experiences related to the participating in the SleepWell trial and therapy, in the context of seeking help for sleep disruption and other mental health problems. The overall narrative was clarified through discussions with supervisors and presentations to the LEAP. |
|  | 10 | Both analytic narratives and data extracts are provided. A reflexive journal was maintained throughout the research process to identify the values, assumptions, and norms in which the research team are embedded. This reflective process captures part of the process of shaping the analytic narrative. |
| **Overall** | 11 | Throughout the analysis extensive time was invested by peer researchers and the lead researcher to consider each transcript individually, the data as a whole, and to revisit each transcript in an iterative process incorporating discussion with other researchers and the LEAP. |
| **Written report** | 12 | The theoretical position and assumptions are clearly stated. The approach to the analysis is detailed, including the use of peer methods. |
|  | 13 | The method and analysis are consistent with the approach., including the critical realist and contextual epistemological position. |
|  | 14 | The language and concepts used in the report are consistent with the critical realist ontological and contextual epistemological positions of the analysis. |
|  | 15 | All researchers were active in the research process and are positioned as such in the report. |
