# Supplementary material for: Sleep disruption and its psychological treatment in young people at risk of psychosis: A peer methods qualitative evaluation
Source: Br J Clin Psychol. 2025 Jul 6;65(3):746–61. doi: 10.1111/bjc.70002 (PMC13456408; doi:10.1111/bjc.70002)
Supplement: Supplementary file 3 [file BJC-65-746-s003.docx]

Supplementary table 1: Additional illustrative quotes

| **Theme 1: The challenge to access mental health treatment; "Bouncing between services"** | |
| --- | --- |
| My experience with the other services was next to none. I went to my local out of hours and they just told me to take off days off and see how I feel. I told my GP I’m feeling pretty suicidal at the moment and they said, “can you hold on two weeks so we can get somebody in to talk about your mental health”. I talked to them about it, said, “I don’t know what’s going on with me and I think I need some therapy for somebody to find out and possibly medication, something. I need something”. I kept on saying that I really wanted something—anything. I had to book in an appointment, I couldn’t get through to my GP. So, I genuinely felt then like OK, well I’ve been abandoned by them as well. | P14 |
| I had a very big issue, obviously, the mental health services didn’t really want me because I don’t fit their criteria, and obviously I was supposed to wait for another programme to open. I don’t even know if the other programme’s opened because no one’s contacted me about it. So, I just kind of needed something because I wasn’t in a good head space, so it was a bit… Uh, to be fair, when I first got told about SleepWell, I thought, ‘That’s quite a strange thing to stick me on, considering,’ but I was okay, but what can I… But I can’t lose anything | P3 |
| I-- like cause I was doing a lot of like-- there was a lot of teams I was working with, with and that and unfortunately they couldn’t help. | P16 |
| **Theme 2: Sleep problems and mental health difficulties are intertwined: “An obvious link”** | |
| Well, obviously when you don’t sleep very well at all, then you feel more drained and more tired and sad because you don’t have the energy for things in your general life. Whereas, when you actually do get a good night’s sleep, then you have that energy to be able to participate in everything you want to. | P11 |
| I felt like I was trapped because I was in this constant state of low energy because of how horrible my sleep was. … I didn’t feel like me at all. I felt like I just completely lost who I was. I felt genuinely empty. I thought I’d been like that before, but no, no this was a whole other animal. | P14 |
| So, in the day, I was just so overwhelmed and anxious by everything, that all I wanted to do was just sleep.  But then, sometimes, it would be the opposite.  I would be so anxious and worked up that I just can’t sleep at all, or I will go to sleep and wake up anxious.  Go back to sleep, wake up anxious and in a cycle.  So, it’s not always that I can’t sleep but sometimes I think I just want to sleep all the time if I’m particularly anxious, which then, I guess, if I’m sleeping in the day because I’m anxious, then at night you can’t sleep because I’ve slept all day. | P13 |
| *Sub theme:* *Bidirectional interaction between sleep problems and psychotic experiences* |  |
| For me, if I get a really bad night of sleep, I can end up being very angry in the morning, very down and if I get a good night of sleep, I’m very happy, very upbeat in the mornings and throughout the day I will be all good. In terms of when it comes to bad nights of sleep, I end up being very slow, very sluggish and very just down on that day. Yeah, during bad nights of sleep, if you haven’t slept well at all, you can end up hearing things that are not there or seeing things that aren’t really there. … I think it’s just the matter of your brain hasn’t had time to chill out, hasn’t had time to reset itself that day and it’s … since been continually working, it’s overworked itself, if that makes sense? | P10 |
| So, I think, cause when I’m going to sleep I’m someone who likes silence. Because the voices were that noise, just irritating or that’s really what, and also the things they were saying would make me spiral and think but obviously first of all it was just having a noise in your head, it’s like having someone with a microphone in your ear which is very hard to sleep with. And then also when I had the voices I then, this is going to sound very sad, but then get scared so then I want to turn the light on cause I’m in the dark and I don’t want to be in the dark when I’ve got voices and I can’t see anything. So, then I have the light on and obviously further disrupts the then going, the being able to go back to sleep. | P15 |
| **Theme 3:** **Flexibility in therapy delivery and provision matters: “Tailored to me as a person”** |  |
| *Sub theme:* *Flexibility and rhetoric matter* |  |
| there was no miscommunication, which commonly happens when you’re like… talking. But I think you guys were so good at making sure of what I was saying, like, you would double check what I was saying and then if you didn’t understand you would ask me to explain again. And I think that’s really good, ‘cause some people just like… leave it. | P2 |
| what I liked about the trial was every week it would be just one or two things like ‘do this if you can’ y’know…‘do this if you want to’ or y’know ‘it would help if you were able to do this’ uhm, it was very, as much as it was laid back I thought it was very informative in the way that all the information was given out subjectively week by week and it was never too much to do. ... This was very tailed to me as a person, y’know if that didn't work, then y’know, this might and I'd try it and go ‘ah yeah it worked!’ Or ‘it didn't ‘ and it would be very self-orient – self-oriented from me which I thought was amazing as well. | P6 |
| It was easy to understand better the different problems which made the cycle up and then how to break them by tackling each one of them.  Because as soon as one was tackled out, the other three would stop. | P9 |
| *Sub theme:* *Active engagement and ownership: making the most of therapy* |  |
| I always, like, pushed myself to attend. Um. I dunno, the biggest thing is, obviously, no one else is gonna change or help you other than, really, you. Like, you can have the tools and stuff, and the therapist and people that are there that really wanta help, but, like, you’re not going to get any better if you’re not trying yourself. | P3 |
| I thought it was really, really helpful to see the actual progress that I was making because obviously it’s more difficult to see it when you’re just doing it. Whereas, if you just fill out a sheet that takes like a minute, and then look back at the one from last week, then you can actually see what’s changed in that last week. Well, it showed me that it was working. So, that was really, really helpful. So, that made me want to persevere a bit more and see it out through to the end. That way I knew that I was actually going to get the best out. | P11 |
| While I was doing the actual therapy, it was … I was sticking to it and it just kind of stuck.  Now it’s just quite normal now. | P7 |
| **Theme 4:** **Improving sleep leads to wider benefits: "Fixing the sleep helped everything else"** |  |
| Once I started to sleep better, and sleep throughout the night, then I had more energy to do things that I wanted to do. I could go out and see my friends and I wouldn’t feel exhausted all the time. And I could actually be a part of what was around me, instead of just feeling like I could … just like walking through fog. That’s almost what it felt like when I wasn’t sleeping properly. Whereas now I just feel like a normal human being. So, that’s a relief. I definitely felt happier, and I think I was worrying a little bit less because I was able to be a part of what was going on around me. I wasn’t just stuck in my own head. So, I was actually listening to what people were saying and actually being able to be involved in conversations fully. | P11 |
| I’m sleeping through the night and I’m being able to sleep when I get into bed. I think my mum’s noticed that I’m not getting up throughout the night. I’m a lot less tired. Um… Just… making things a lot easier. I’m a lot less stressed. | P1 |
| I think it definitely reduces stress because you’re not waking up stressed, you’re not waking up like, “I didn’t sleep properly” and starting the day on bad terms, which makes it a lot easier to get on with the day while you’ve got energy and feel refreshed.  So, it’s definitely a stress-reliever and you start the day on good terms.  And I think that already was better than how I was before when I would wake up and be like, “I don’t feel good.  The day is a bit rubbish.”  When I first started SleepWell, I was having those, like, hearing things and seeing things.  That sort of faded.  I’m not sure if that’s to do with sleep or just in general.  But that sort of went away.  But the anxiety side and the low mood side has definitely been fixed due to the sleep improvement. | P8 |
