# Supplementary material for: Sleep disruption and its psychological treatment in young people at risk of psychosis: A peer methods qualitative evaluation
Source: Br J Clin Psychol. 2025 Jul 6;65(3):746–61. doi: 10.1111/bjc.70002 (PMC13456408; doi:10.1111/bjc.70002)
Supplement: Supplementary file 4 [file BJC-65-746-s004.docx]

**Participant theme contributions**

Supplementary table 2. Summary of participant contributions to each themes

| **Themes** | | **Participants** | | | | | | | | | | | | | | | |
| --- | --- | --- | --- | --- | --- | --- | --- | --- | --- | --- | --- | --- | --- | --- | --- | --- | --- |
|  |  | **1** | ***2*** | **3** | ***4*** | ***5*** | **6** | **7** | **8** | **9** | ***10*** | **11** | ***12*** | ***13*** | **14** | **15** | **16** |
| 1) The challenge to access mental health treatment: "Bouncing between services" | |  |  | X |  |  | X | X | X | X | X | X |  | X | X | X | X |
| 2) Sleep problems and mental health difficulties are intertwined: “An obvious link” | | X | X | X |  | X | X | X | X | X | X | X | X | X | X | X | X |
| Bidirectional interaction between sleep problems and psychotic experiences | | X |  | X |  |  | X |  | X |  | X |  |  |  |  | X | X |
| 3) Flexibility in therapy delivery and provision matters: “Tailored to me as a person” | | X | X | X | X |  | X | X | X | X | X | X | X | X | X | X | X |
|  | Flexibility and rhetoric matter | X | X | X | X |  | X |  | X | X | X | X |  |  | X | X | X |
|  | Active engagement and ownership: making the most of therapy | X |  | X |  |  | X | X | X |  |  | X | X | X | X | X | X |
| 4) Improving sleep leads to wider benefits: "Fixing the sleep helped everything else" | | X |  | X |  |  | X | X | X | X | X | X |  |  | X | X | X |

*Note.* ***Italicised*** *participant numbers did not receive therapy within the trial*
